# Aortic Valve Plasty for Coexistence of Discrete Subvalvular Aortic Stenosis and Quadricuspid Aortic Valve: A Case Report

**DOI:** 10.7759/cureus.75491

**Published:** 2024-12-10

**Authors:** Masato Saitoh, Takuma Yamasaki, Tomoaki Tanabe, Shuichi Tochigi, Imun Tei

**Affiliations:** 1 Cardiovascular Surgery, Ayase Heart Hospital, Tokyo, JPN

**Keywords:** aortic regurgitation, aortic valve plasty, cardiac surgery, discrete subvalvular aortic stenosis, quadricuspid aortic valve

## Abstract

Subvalvular aortic stenosis typically manifests at a young age and rarely presents in adulthood. It may cause left ventricular outflow tract stenosis, which requires surgical treatment in severe cases. The coexistence of discrete subvalvular aortic stenosis and quadricuspid aortic valve is a highly unusual finding. We present a case of a patient with coexisting discrete subvalvular aortic stenosis and quadricuspid aortic valve, who underwent aortic valve plasty and subvalvular aortic stenotomy. A 63-year-old woman with a history of shortness of breath on exertion was referred to our hospital after being diagnosed with discrete subvalvular aortic stenosis and quadricuspid aortic valve at another hospital. Echocardiography revealed membranous tissue below the aortic valve and a quadricuspid aortic valve. Dobutamine stress echocardiography showed a mean pressure gradient of 75 mmHg and Vmax of 5.9 m/s in the subaortic membranous area. The subaortic valve membranous structures were first resected during surgery to release the subaortic stenosis. The aortic valve had four cusps with an accessory cusp between the right and left coronary cusps. Next, the right coronary cusp was sutured to the accessory cusp and converted to a single valve. Intraoperative transesophageal echocardiography showed trivial aortic regurgitation. The intraoperatively resected subvalvular tissue contained fibrous connective tissue with fibrous thickening and mucinous degeneration. At one-year postoperative follow-up, there is no subvalvular aortic stenosis and aortic regurgitation recurrence, and the patient is doing well. Long-term outcomes of aortic valvuloplasty for quadricuspid aortic valves are not well-characterized in the literature. Owing to the high relapse rate of subvalvular aortic stenosis, rigorous follow-up with echocardiography every six to twelve months is essential to evaluate the long-term success of aortic valve plasty.

## Introduction

The reported prevalence of quadricuspid aortic valve in autopsy cases is 0.008%-0.033% [1]. The reported probability of a quadricuspid aortic valve in patients undergoing aortic valve surgery is 1.43%-1.46% [2,3]. Subvalvular aortic stenosis typically manifests at a young age and rarely in adulthood. Structures below the valve can cause left ventricular outflow tract obstruction, and severe cases may require surgical treatment. Subvalvular aortic stenosis can be complicated by congenital anomalies such as ventricular septal defect, bicuspid aortic valve, coarctation of the aorta, and patent ductus arteriosus [4,5]. There are no reports investigating the prevalence of aortic quadricuspid valve coexisting with discrete subvalvular aortic stenosis, and its incidence remains unknown. Aortic regurgitation is the most common complication associated with the quadricuspid aortic valve. Discrete subvalvular aortic stenosis and quadricuspid aortic valve coexistence is an exceedingly rare clinical entity. To date, only one case has been reported in the literature, and there have been no recent reports [6]. We present a case of a patient with coexisting discrete subvalvular aortic stenosis and quadricuspid aortic valve, who underwent aortic valve plasty and subvalvular aortic stenotomy.

## Case presentation

A 63-year-old woman had a history of shortness of breath on exertion since her youth, but she had not visited a hospital. She visited her local doctor in December 2015 after an abnormal electrocardiogram was noted during a physical examination. She was being followed up with a diagnosis of discrete subvalvular aortic stenosis and quadricuspid aortic valve but was referred to our hospital in March 2023 due to the relocation of her doctor. She presented to our hospital with severe shortness of breath on exertion. An echocardiogram showed accelerated blood flow in the left ventricular outflow tract (LVOT) with a Vmax of 3.6 m/s. After obtaining patient consent, a decision was made to perform surgery. The patient was receiving medication for hypertension and dyslipidemia for an extended period.

**Figure 1:**
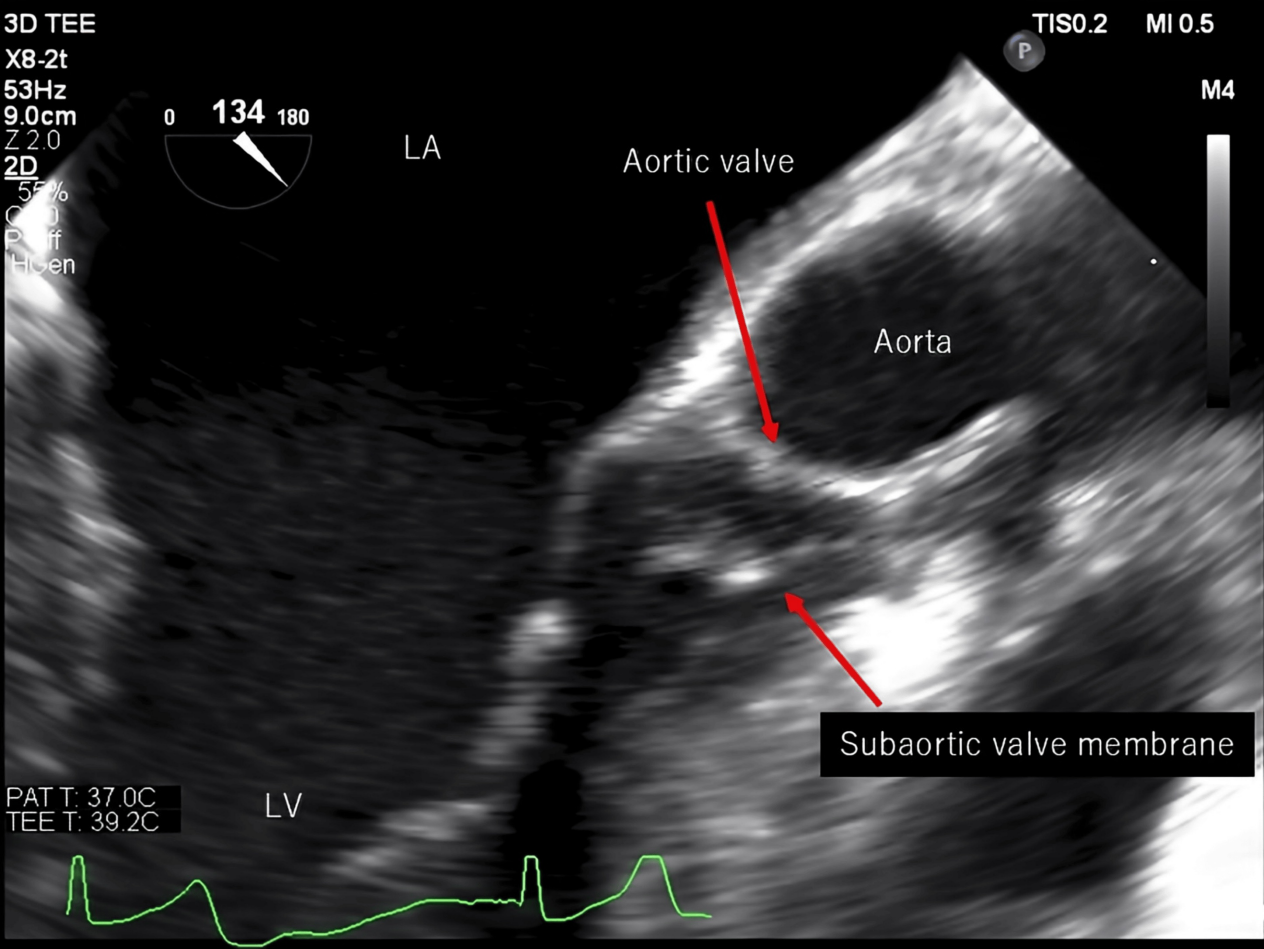
Transesophageal echocardiogram A membrane structure was identified below the aortic valve.

On admission, the patient’s height and weight were 160.8 cm and 75 kg, respectively. Her blood pressure was 121/60 mmHg, heart rate was 59 bpm (sinus rhythm), and SpO2 was 97%. A systolic murmur was audible in the left sternal border in the 4th intercostal space. Her brain natriuretic peptide level was elevated (74.5 pg/mL) and her estimated glomerular filtration rate was decreased (55.7 mL/min). Chest radiography revealed a cardiothoracic ratio of 52.4% with no signs of pulmonary congestion or pleural effusion. Heart failure was well controlled. Transthoracic echocardiography showed a subaortic thin membrane located 9 mm below the aortic valve. A similar finding was also observed in transesophageal echocardiography (Figure 1).

**Figure 2:**
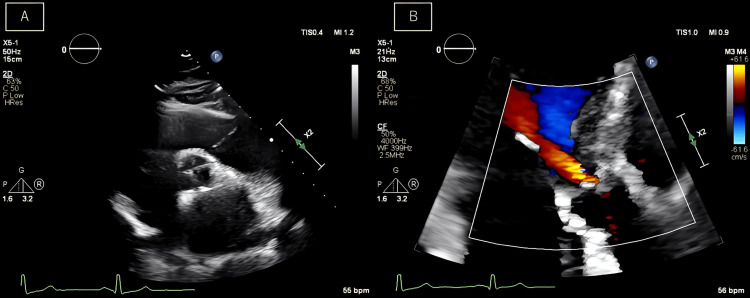
Preoperative transthoracic echocardiogram (A) Preoperative echocardiogram showing a quadricuspid aortic valve. (B) The aortic regurgitant jet is not vertical, but ubiquitously depicted toward the mitral cusp.

The velocity of the subaortic membranous area was measured using pulsed Doppler echocardiography during dobutamine stress echocardiography. The mean pressure gradient of 75 mmHg (normal range: <25 mmHg) and Vmax 5.9 m/s (normal range: < 2.5 m/s) in the subaortic membranous area. The dimensions of cardiac cavities were normal, and the left ventricle showed normal contractility and systolic function. The aortic valve had four cusps, and the aortic regurgitant jet reached as far as the mitral valve leaflet. A qualitative assessment revealed mild regurgitation (Figure 2A). The aortic regurgitant jet was not vertical but ubiquitously depicted toward the mitral cusp. The aortic regurgitant jet interfered with the subvalvular stenosis, making accurate qualitative assessment and quantitative assessment difficult (Figure 2B). Coronary computed tomography revealed no coronary artery stenosis.

**Figure 3:**
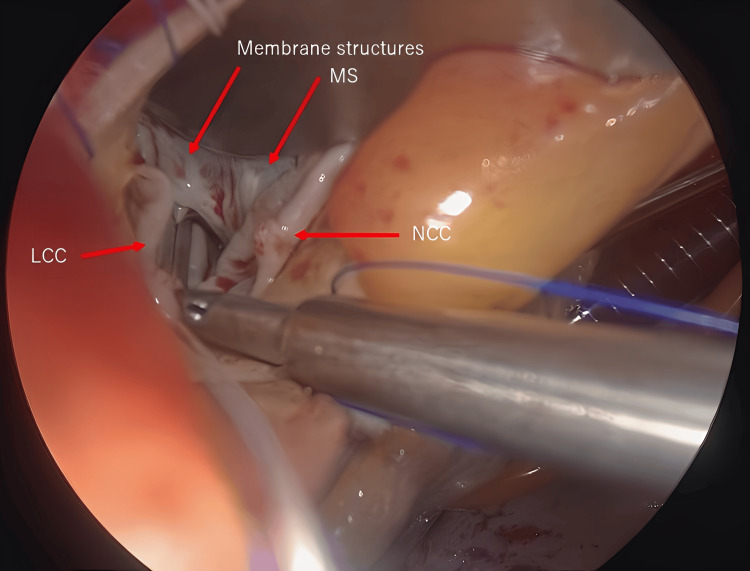
Intraoperative subaortic valve Photograph showing a membranous structure below the right coronary cusp.

The surgery was performed via a median sternotomy. Cardiopulmonary bypass was established as usual, with cannulation of the ascending aorta and superior and inferior vena cava. After clamping the ascending aorta, myocardial protection was achieved with antegrade cardioplegic administration. The ascending aorta was transected 1 cm above the right coronary artery. A membranous structure approximately 1 cm below the right coronary cusp was resected along with the myocardium of the ventricular septum to release the subaortic stenosis (Figure 3).

**Figure 4:**
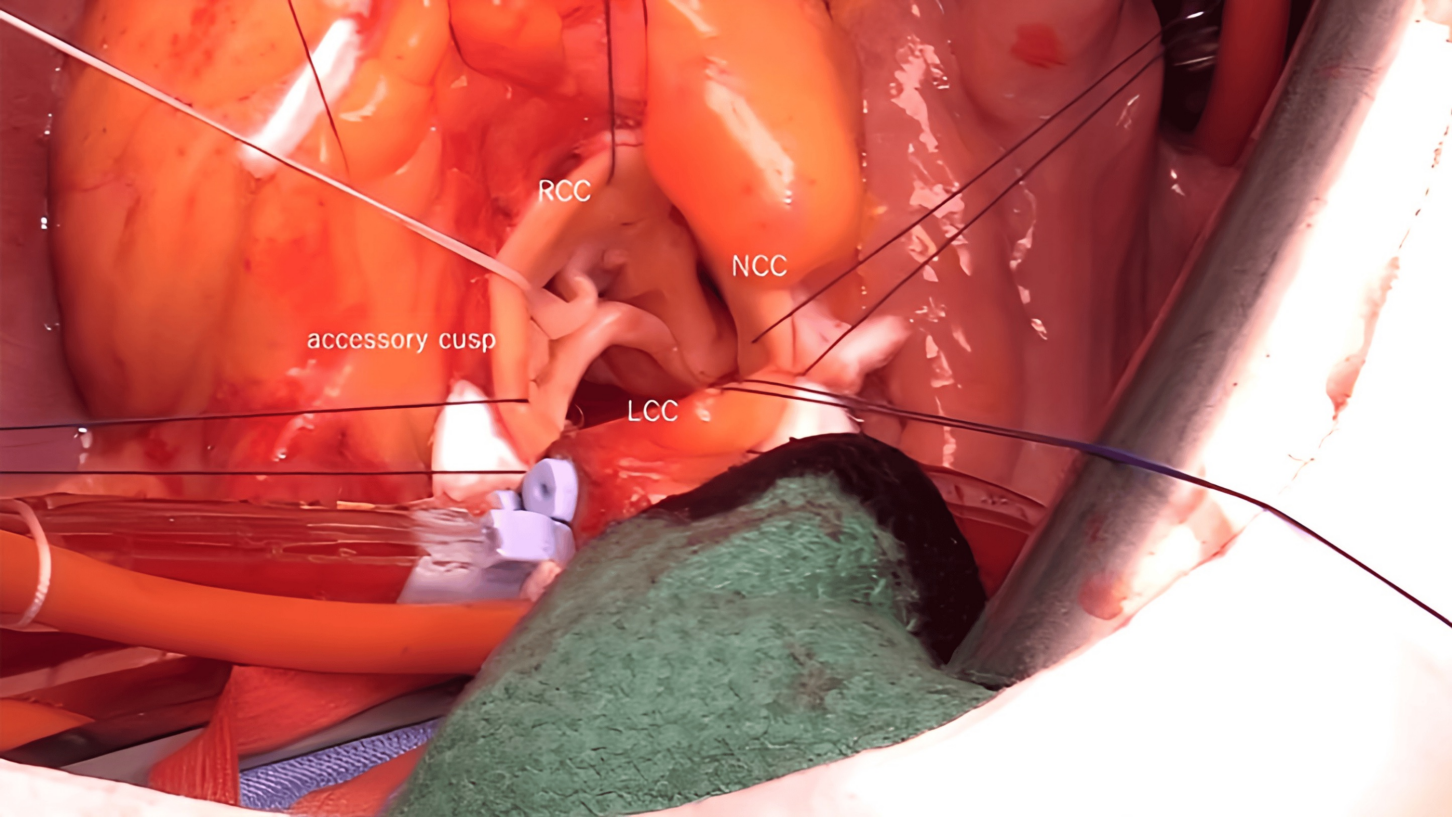
Intraoperative photograph showing a quadricuspid aortic valve A small accessory cusp is seen between the right and left coronary cusps.

Observation of the aortic valve revealed four cusps. An accessory cusp was observed between the right and left coronary cusps (Figure 4).

**Figure 5:**
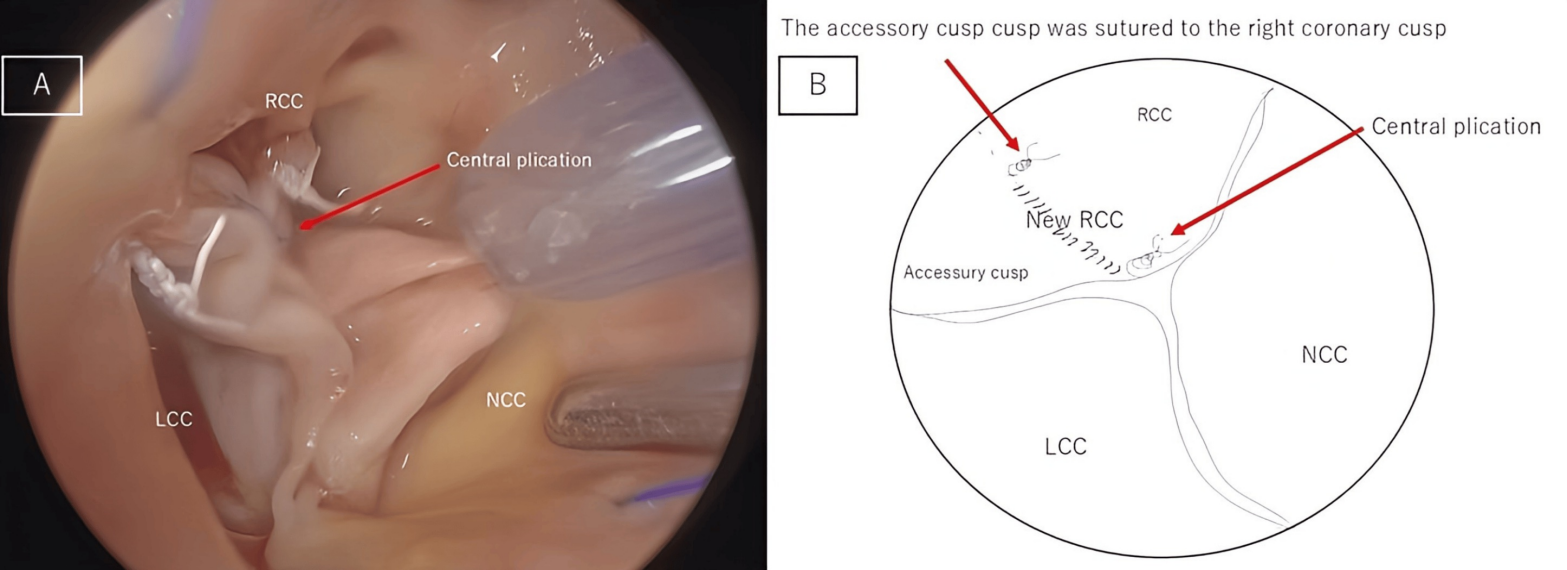
Photograph obtained after aortic valve plasty (A) intraoperative photograph, (B) schematic diagram. The accessory cusp and the right coronary cusp have been sutured to form a single valve. Central plication has been performed on the right coronary cusp.

Cusp measurements were taken using dedicated cusp calipers (Genesee BioMedeical, Denver, USA). The accessory cusp's effective height (eH) was 6.0 mm and the coaptation height (cH) was 3.7 mm, smaller than the other valve cusps, which was the likely cause of regurgitation. The eH of the right coronary apex was 9.8 mm and the cH was 6.5 mm. The right coronary cusp was sutured to the accessory cusp and converted to a single valve. Central plication was performed on the right coronary cusp using 6-0 polypropylene sutures to balance the height of the left coronary cusp and the noncoronary cusp (Figure 5A, 5B).

**Figure 6:**
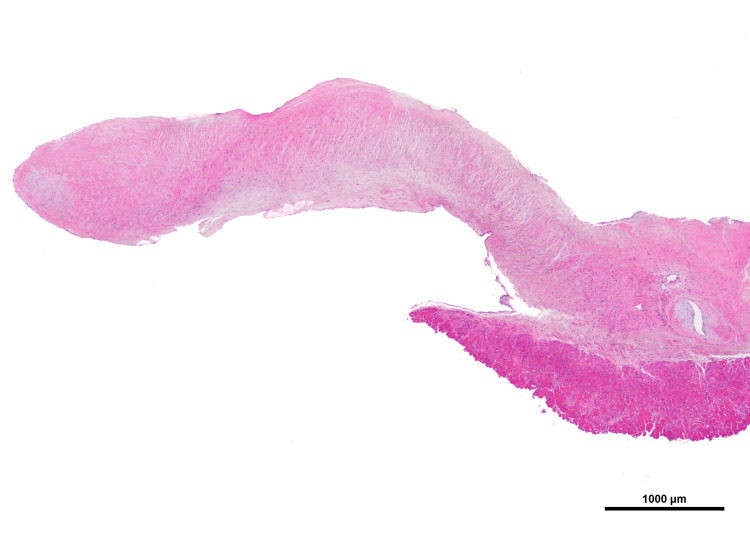
Hematoxylin and eosin staining of resected subvalvular tissue Fibrous thickening was observed in the membranous portion.

After central plication, the eH of all three cusps was >9 mm. After the suture closure of the aortotomy, the patient was weaned off cardiopulmonary bypass, and the operation was completed. Intraoperative transesophageal echocardiography showed trivial aortic regurgitation. The operative time was 187 min, the cardiopulmonary bypass time was 104 min, and the cross-clamp time was 69 min. Histopathological examination of the resected subvalvular tissue showed fibrous connective tissue with fibrous thickening and mucinous degeneration (Figure 6).

**Figure 7:**
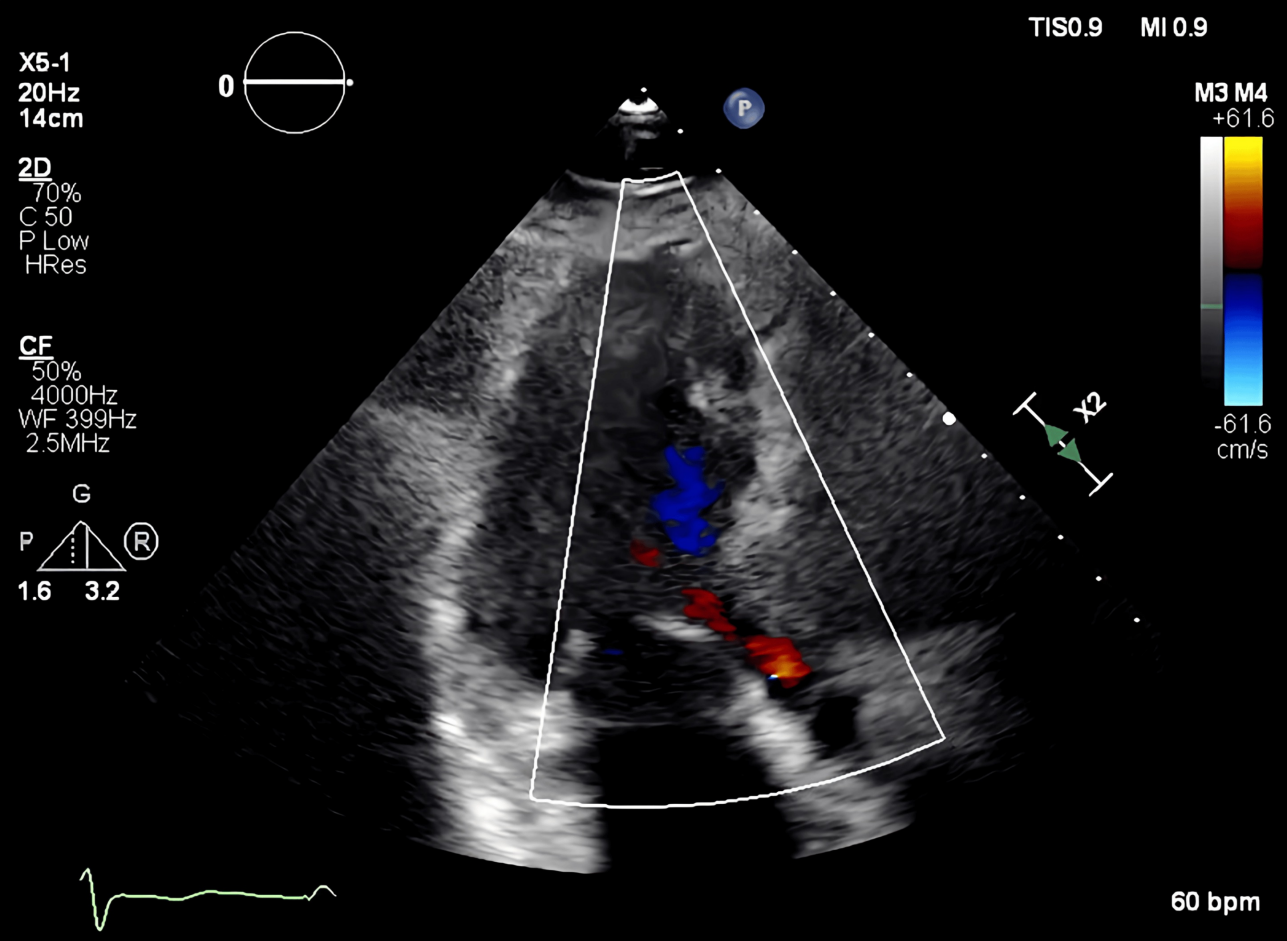
Follow-up echocardiogram Follow-up echocardiogram 1 year after surgery shows no signs of recurrence of subvalvular aortic stenosis and aortic regurgitation.

There was no calcification, deposits, or granulation. Postoperative transthoracic echocardiography showed no aortic valve regurgitation or accelerated blood flow in the LVOT. The Vmax in the LVOT was 2.6 m/s. She was discharged on the 16th postoperative day. As of the one-year postoperative follow-up, there is no subvalvular aortic stenosis and aortic regurgitation recurrence, and the patient is doing well (Figure 7).

## Discussion

The structure of subvalvular aortic stenosis is most commonly described as a fibromuscular ring of tissue, but can also present as an incomplete shelf or ridge-like structure [7-9]. The stenotic tissue typically consists of endothelium, an acid mucopolysaccharide-rich subendothelial layer, a collagen-rich fibrous layer, a fibroelastotic layer, and a smooth muscle layer [10]. In this case, too, there was a membrane-like substance with an annular structure below the aortic valve, which had caused subvalvular aortic stenosis. Histopathological examination of the resected membranous material showed fibrous connective tissue, typical of discrete subvalvular aortic stenosis. Patients with subvalvular aortic stenosis may be asymptomatic or experience fatiguability, dyspnea, palpitations, chest pain, and syncope. The disease typically progresses slowly. In this case, discrete subvalvular aortic stenosis coexisted with quadricuspid aortic valve. Although the quadricuspid aortic valve is infrequently associated with congenital heart disease, it is reported to be complicated by coronary artery anomalies such as the anomalous origin of the coronary artery, hypoplastic coronary artery, and single coronary artery in 30% of cases [11]. The reported incidence of aortic regurgitation in patients with quadricuspid aortic valves is 47%-93% [12,13]. Theoretically, aortic regurgitation in the quadricuspid aortic valve occurs due to the disproportion of the cusps, which may result in abnormal valve motion mechanisms leading to early fibrotic changes of the leaflets, thus aggravating valve dysfunction [13]. In the present case, the aortic regurgitant jet was displaced by structures below the aortic valve, making accurate assessment of aortic regurgitation difficult. There were no coronary complications and the symptoms were believed to be caused by aortic regurgitation and progressive subvalvular aortic stenosis. Releasing the subvalvular aortic stenosis alone in this patient would have resulted in residual symptoms due to aortic regurgitation. The quadricuspid aortic valve is highly likely to progress to aortic regurgitation in the future without intervention, and this case is also considered likely to experience worsening aortic regurgitation.

Therefore, we considered it important to control regurgitation and to distribute stress evenly among the cusps before the degeneration of the native aortic valve. Morphologically, we anticipate that fusion of the right coronary cusp and accessory cusp will result in an effective height of at least 8 mm and a coaptation height of at least 4 mm. We considered that aortic valve plasty could control regurgitation. This case corresponded to Hurwitz type B, and the right coronary cusp and accessory cusp, considered to be the cause of the present regurgitation, were sutured together to form a single cusp [14]. Central plication was performed so that the eH of the formed RCC was at least 8 mm. Intraoperative transesophageal echocardiography showed trivial aortic regurgitation and no additional procedures were required. Long-term outcomes of aortic valvuloplasty for bicuspid and tricuspid valves have been reported, with the Homburg group reporting a 5-year reoperation-free rate of 88% for bicuspid valves and 97% for tricuspid valves [15]. However, the long-term outcomes of aortic valvuloplasty for quadricuspid aortic valves are unknown. Song et al. reported good mid-term results with bovine pericardial tricuspidization and sinotubular junction (STJ) fixation with the inner ring for quadricuspid aortic valves [16].

There are a few case reports of aortic valvuloplasty for quadricuspid aortic valve [16-18], but only a limited number of reports of tricuspidization using only autologous valve leaflet tissue. Masse et al. reported that recurrence, which occurs in 8-34% of patients over a 10-year period, has been associated with young age at both initial diagnosis and surgical intervention, smaller aortic annulus, the proximity of the obstruction to the aortic valve, and a higher preoperative peak LVOT gradient. Females have a 1.5 times greater risk of recurrence compared to males. This increased risk could be associated with the smaller LVOT anatomies in women, but the role of gender in DSS recurrence has not been investigated [10]. This case meets two risks, we must carefully observe the success or failure of the aortic valve valve plasty and subvalvular aortic stenotomy, as well as the postoperative course.

## Conclusions

We present a case of a patient with coexisting discrete subvalvular aortic stenosis and quadricuspid aortic valve, who underwent aortic valve plasty and subvalvular aortic stenotomy. One year later, echocardiography showed no recurrence of membranous structures or increase in the LVOT tract pressure gradient. The long-term outcomes of aortic valve plasty for quadricuspid aortic valve remain uncertain, necessitating close follow-up. The postoperative course was uneventful.
